# Complete Genome Sequencing of Mycoplasma synoviae Strain HN01, Isolated from Chicken in Henan Province, China

**DOI:** 10.1128/MRA.01480-19

**Published:** 2020-02-06

**Authors:** Bin Xu, Rui Liu, Lihan Tao, Meijuan Ding, Fengying Lu, Jingfeng Zhang, Huawei Sun, Chuanmin Liu, Qunxing Pan, Sha Zhao, Xiaofei Zhang

**Affiliations:** a Key Laboratory of Veterinary Biological Engineering and Technology of Ministry of Agriculture, Institute of Veterinary Medicine, Jiangsu Academy of Agricultural Sciences, Nanjing, China; b National Center for Engineering Research of Veterinary Bio-products, Jiangsu Academy of Agricultural Sciences, Nanjing, China; Queens College

## Abstract

Here, we report the complete genome sequence of Mycoplasma synoviae HN01, a virulent epidemic strain isolated from a sick chicken with synovitis in Henan Province, China. HN01 is the Asian source of an M. synoviae strain that is completely sequenced, genome annotated, and published with relevant data.

## ANNOUNCEMENT

Mycoplasma synoviae is a major avian pathogen. It causes serious harm to poultry farming. Its infection can cause respiratory damage, synovitis, osteoarthritis, growth retardation, and decreased egg production in chickens, turkeys, and other poultry worldwide (1, 2). Here, M. synoviae was cultured in modified Frey’s broth or agar at 37°C with 5% CO_2_, as described previously (3). M. synoviae HN01 was isolated from a sick chicken with synovitis in Henan Province, China. Identification was confirmed by sequencing the PCR products of the 16S rRNA genes. Genomic DNA (gDNA) was extracted using a mycoplasma gDNA minikit (Biomiga, China), according to the manufacturer’s instructions.

Genomic DNA was sequenced at the Beijing Genomics Institute (BGI) in Shenzhen, China. For long-read sequencing, genomic DNA was fragmented to 10 to 20 kbp using a g-TUBE apparatus (Covaris). The sequencing library was constructed using the SMRTbell template prep kit 1.0 according to the PacBio 20-kb library protocol. Sequencing was performed on the PacBio RS II instrument in one single-molecule real-time (SMRT) cell v3. There were 55,866 polymerase reads after sequencing. The total bases, mean length, and *N*_50_ value of the polymerase reads were 933,278,509 bp, 16,705 bp, and 24,040 bp, respectively. The average quality of polymerase reads was 0.85. The polymerase reads were filtered to remove adaptor sequences, sequences under 1,000 bp in length, and sequences with a quality of less than 0.80 to obtain more accurate and reliable subreads. There were 96,823 filtered subreads, with a total base count of 930,261,090 bp. The average length of the subreads was 9,607 bp, with an *N*_50_ value of 11,503 bp. The average quality of the subreads was 0.85.

For short-read sequencing, genomic DNA was fragmented using an AFA-TUBE apparatus (Covaris). The paired-end sequencing library, which was constructed with 270-bp inserts, was prepared using a NEBNext Ultra II DNA library prep kit (New England BioLabs, UK) and sequenced using the Illumina HiSeq 4000 platform. A total of 104 Mb raw data containing 699,870 150-bp reads were produced. After filtering out low-quality reads with SOAPnuke using default parameters (4), we obtained 96 Mb clean short reads.

The genome was assembled with the Celera assembler 8.3 and FALCON 0.3.0 (5, 6). Quiver in SMRT analysis v2.3.0, GATK v1.6-13 (https://www.broadinstitute.org/gatk/), SOAPsnp (7), and SOAPindel (8) were used for single-base correction of the genome. Default settings were used for all software used. The consensus assembly generated one contig of 817,087 bp (1,270-fold coverage), representing the HN01 genome. The G+C content of the genome was 28.32%.

Gene prediction and functional annotation were processed using the GeneMarkS+ annotation system in the NCBI Prokaryotic Genome Annotation Pipeline (9–11). A total of 738 genes were predicted, including 656 protein-coding genes, 44 RNA genes (34 tRNAs, 7 rRNAs, and 3 noncoding RNAs), and 38 pseudogenes.

**FIG 1:**
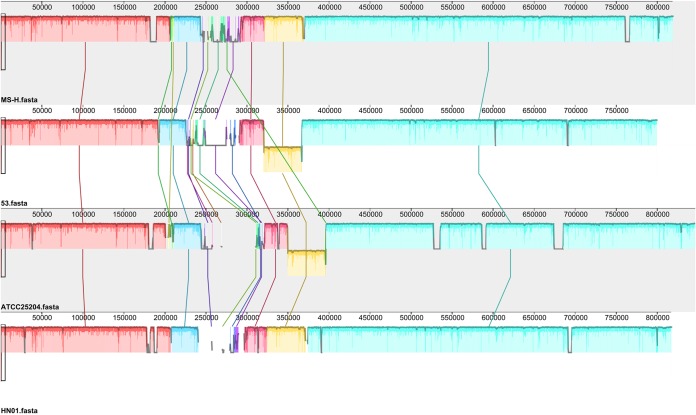
Genomic sequence alignment of M. synoviae strains MS-H, 53, ATCC 25204, and HN01. The alignment was generated using Mauve software with default settings.

The three M. synoviae strains for which the whole-genome sequences are now listed in GenBank are the North American source standard strain ATCC 25204 (accession number NZ_CP011096.1), the South American Brazilian source field strain 53 (NC_007294.1), and the Australian source attenuated vaccine strain MS-H (NZ_CP021129.1). Whole-genome sequence alignments were performed using Mauve software (http://darlinglab.org/mauve/mauve.html). Results revealed that the chromosomes of MS-H, 53, ATCC 25204, and HN01 were colinear (Fig. 1).

### Data availability.

The complete genome sequence and annotation of M. synoviae strain HN01 have been deposited in GenBank under accession number CP034544. The raw data were deposited in the Sequence Read Archive (SRA) database under the accession numbers SRR10270520 (PacBio RS II) and SRR10270519 (Illumina). The BioProject accession number is PRJNA509945. The BioSample accession number is SAMN10591532.
